# Supplementary material for: Hybrid Immunity Shifts the Fc-Effector Quality of SARS-CoV-2 mRNA Vaccine-Induced Immunity
Source: mBio. 2022 Aug 24;13(5):e01647-22. doi: 10.1128/mbio.01647-22 (PMC9600672; doi:10.1128/mbio.01647-22)
Supplement: TABLE S1 [file mbio.01647-22-s0001.pdf]

Table S1

Term	Variable	Effect Type	Number of Terms
$(\log MFI)_{ijk}$	Sample measurement for a single feature		
μ	Mean log(MFI) for that feature	Fixed	1
g_i	Group effect of interest (dose and infection status)	Fixed	4 groups (V1/V2 x infected/not)
b_j	Batch main effect	Random, $b \sim N(0,\ \sigma_b^2)$	2 batches
$(gb)_{ij}$	Group x batch interaction	Random, $(gb) \sim N\left(0,\ \sigma_{gb}^2\right)$	4 groups x 2 batches = 8
ϵ_{ijk}	Residual error	Random, $\epsilon \sim N(0,\ \sigma^2)$	
